# Chicken or the Egg: The Reciprocal Association Between Feeding Behavior and Animal Welfare and Their Impact on Productivity in Dairy Cows

**DOI:** 10.3389/fvets.2018.00305

**Published:** 2018-12-05

**Authors:** Pol Llonch, Eva Mainau, Ignacio R. Ipharraguerre, Fernando Bargo, Gemma Tedó, Marta Blanch, Xavier Manteca

**Affiliations:** ^1^School of Veterinary Science Autonomous University of Barcelona, Cerdanyola del Vallès, Spain; ^2^Innovation Division, Lucta S.A. UAB Research Park, Cerdanyola del Vallès, Spain

**Keywords:** dairy cattle, feeding behavior, feed efficiency, productivity, welfare

## Abstract

Feeding behavior in dairy cattle has a significant impact on feed efficiency, which is important for increasing the profitability of livestock and, at the same time, reducing the environmental impact. Feeding behavior can be measured by feeding time, meal duration, meal frequency, feeding rate, and rumination time. Higher feed intake is related to lower feed efficiency; whereas, an increase in feeding time facilitates chewing, reduces feed particle size and increases its digestibility. More frequent and shorter meals are usually associated with a more efficient use of feed due to improvement of feed digestibility. Rumination time is positively associated with milk production. Impaired health is associated with variations in feeding behavior, which can be used to identify and predict some diseases such as ketosis, mastitis, or lameness. Changes in rumination time are also a reliable indicator of mastitis, lameness, ketosis, abomasal displacement, and the onset of calving. In addition to the cause-effect relationship between disease and changes in feeding behavior, there are also some cases in which changes in feeding behavior may lead to an increased risk of disease, as exemplified by the relationship of feeding rate with sub-acute ruminal acidosis. Feeding behavior is regulated by internal and external factors and some of them are relevant for animal welfare. The main welfare-associated factors influencing feeding behavior are social behavior and temperament, and environmental effects. Cattle are social animals and hierarchy has a notable impact on feeding behavior, especially when access to feed is limited. Competition for feed causes a reduction in the average feeding time but increases feeding rate. Excitable animals visit the feeder more often and spend less time per meal. High environmental temperature affects feeding behavior, as heat-stressed cattle change their feeding pattern by concentrating the feeding events in crepuscular hours, leading to an increased risk of sub-acute ruminal acidosis. In conclusion, feeding behavior is a determinant feature for improving efficiency, productivity and welfare of dairy cattle. Routine assessment of feeding behavior allows monitoring of health and production status of dairy cattle at the individual and farm level, which is a useful tool to optimize the management of livestock.

## Introduction

The study of feeding behavior in dairy cattle has received notable attention for its association with productivity and efficiency (1). Improved feed efficiency in dairy cattle is important because of its economic value, but also because of the increasing need to reduce waste associated with animal production (e.g., manure and enteric methane) and its impact on the environment. Because of the increased farm profitability and the reduced environmental impact associated with better efficiencies, research has devoted much effort toward studying strategies to improve feed efficiency. Besides productivity, feeding behavior has a bidirectional association with animal welfare. Some animal welfare-related problems, such as impaired health or pain, lead to changes in feeding behavior; whereas, changes in feeding behavior can lead to health problems. This review will focus on understanding the factors that regulate feeding behavior and its implications for productivity, while revising the bidirectional association between feeding behavior and dairy cattle welfare.

## Drivers of Cattle Feeding Behavior: Between Appetite and Satiety

In 1985, Le Magnem (2) defined feeding behavior of an animal as the selection and ingestion of nutrients required for growth and maintenance. In ruminants, feeding is a predominant behavior and they spend a large proportion of the day feeding. A high-producing Holstein can eat more than 25 kg of dry matter in a day. The average time devoted to feeding may be as much as 12 h, distributed in several discrete feeding events or “meals” that are separated by periods of time longer than eating events (1). Cattle often show a crepuscular pattern in feeding activity, with clear peaks around dawn and dusk (3). In ruminants, feeding is followed by rumination, where the feed is fermented by ruminal microbiota, to make nutrients accessible. Both feeding and rumination are mutually necessary. However, there is an inhibitory relationship between feeding and ruminating motivations with preference for feeding behavior (4), that in a given scenario of a hungry cow with a need for rumination, she will always first eat rather than ruminate. It is clear that feeding without ruminating may not provide any benefit for the cow but this example is used to illustrate the importance of feeding over other crucial activities.

Appetite refers to the subjective desire to eat, sometimes due to hunger. The most obvious conditions that provoke hunger, and therefore stimulate eating, are energy deficit and weight loss induced by feed deprivation (2, 5). In cattle, feed deprivation or hunger is associated with an increase of ghrelin plasma concentrations which stimulate feed intake (6). In grazing cattle, longer periods of feed deprivation (fasting time) result in longer grazing bouts, higher intake rates and reductions in rumination time during the grazing period. This leads to more pronounced changes in rumen pH and higher rumen load (7).

Satiety is the state during which, from the end of one meal to the motivation for the next, an animal is not stimulated to eat. Satiation is reached after chewing and swallowing a minimum quantity of feed, followed by its accumulation in the stomach and passage through the intestines. The filling during a meal achieves satiety through a negative feedback counteracting the initial motivation of the hunger. It is, therefore, a feedback function at several levels: oral, gastric, and post-ingestive (8). The quantity of feed that is necessary to reach satiety is highly variable and dependent on variables such as age, state of production and breed.

Feeding behavior is not only affected by homeostatic needs, like appetite or satiety, but also by hedonic and motivational factors associated with foods through experiences and expectations of rewards (9).

Motivation for feeding in ruminants is influenced by internal and external factors. External factors include food sensory characteristics and are considered as an incentive that influences feeding behavior in ruminants (9–11). Internal factors, like physiological and metabolic responses, may interact with sensory properties. For instance, energy sources could be sensed by nutrient sensors present in the tongue and along the gastrointestinal tract, like the sweet taste receptor, which is able to trigger hormone secretions that induce changes in satiety (12). To reinforce motivation, taste receptors are also able to trigger hedonic-reward responses through stimulation of opioid receptors from the hypothalamus and limbic structures (13). Changes in either internal or external factors might change the motivation, but nonetheless, none of them should be regarded as dominant (8).

The internal factors regulating feed intake (e.g., physiological stimulation of feed intake as a result of hunger) can be amplified through the positive sensory stimulation or palatability of the feed. Palatability has a major influence on feeding behavior in ruminants, and the sense of taste is highly developed in cattle (14, 15). Palatability has been considered as the interaction between pre- and post-ingestive information and the subsequent learning process, which is influenced by other several factors, such as genetic background, environmental conditions and social context (9, 10, 13, 16–18).

Sensory additives (flavors) are feed additives that could be included in the diet of animals to modulate the feeding behavior, mostly with the objective to enhance the palatability of feed and encourage feed intake. Taste and smell are the main senses associated with feed intake; therefore, flavors are aromatic and/or tasty substances that stimulate these senses and trigger feeding motivation. Feeding stimulation mostly results in higher dry matter intake (DMI) and higher milk yield (19). For instance, Migliorati et al. (20, 21) observed that dairy cows who received a flavored diet (aromatic sweetener) in the automatic milking system were attracted more to the feeder compared to control (number of visits to the feeder was increased). Merrill et al. (22) evaluated a flavor enhancer to improve forage palatability in lactating dairy cows and observed a tendency for greater DMI (+1.5 kg/d) and milk yield (+3.9 kg/d) in multiparous dairy cows.

Social behavior adds to feed components as an external factor regulating feeding behavior. For instance, social facilitation is a feeding motivation influencer, in which an animal's motivation to eat is stimulated by the sight and sound of other animals eating (9). This phenomenon, initially described by Curtis and Houpt (23), suggest that when one cow eats, another is stimulated to eat as well, whether she is hungry or not. Therefore, the management of the feed bunk should consider social behavior when designing the optimal feeding management of the herd. The link between feeding management and social behavior relies on whether cows have free access (i.e., no competition) to feed affecting the total time spent eating and pattern of eating each day. Some authors consider that feeding management regulates eating behavior (24).

## Monitoring Feeding Behavior

Feeding behavior analyses the amount and distribution of feed intake. In 1999, Nielsen (25) proposed a methodology to analyse feeding behavior that can be applied in different species and considers six different measures: dry matter intake (DMI; kg/day), average intake per visit to the feeder (kg/visit), number of visits to the feeder (visits/ day), time spent in the feeder (min/day), average time per visit (min/visit), and feeding rate (FR, g/min). These measures may not be applicable to assess feeding behavior in cattle on pasture. Instead, pasture DMI can be calculated based on the time spent grazing, the biting rate and the bite size, using the following function; pasture DMI = grazing time × biting rate × bite size (26). In 2007, Chapinal et al. (27) validated some of these variables (the frequency and the number of visits to the feeder and the average intake per visit) to study feeding behavior in dairy cattle. Since then, numerous studies have used these parameters to study the association between feeding behavior, productivity and, more recently, animal welfare. For example, in dairy cows the time spent eating daily increases during the first weeks of lactation (28), which confirms the finding of Kertz et al. (29) that the DMI increases progressively at the beginning of lactation for ~9 weeks, showing the relationship between feeding behavior and feed intake.

In recent years, numerous tools have been developed to monitor behavior in cows such as pedometers or electronic collars, which measure activity or feeding behavior, respectively (30). The jaw movement' recorder (IGER) is used to monitor feeding behavior in grazing cattle (31) through a time-stamped record of bites and chews. Measurements commonly used to describe feeding behavior include frequency and duration of meals (25, 27). However, these data may be used to calculate indices like the average duration of meals, which has been proven very useful in predicting rumination changes in ruminal acidosis (32). If the feed intake is also known, it can be used to calculate the FR, which as later discussed in this review, can be directly associated with some health problems.

Sorting behavior has also received the attention of science researchers for its implications on nutrition and health of dairy cattle. Sorting behavior is usually assessed through the comparative analysis and weight between the offered diet and orts (33). Dairy cattle, selectively consume (sort for) the shorter, concentrate particles in their TMR, while selectively refusing (sort against) the longer, forage particles (34). In 1981, Campling and Morgan (35) suggested that cattle are adept at gathering feed using their lips, teeth, and tongue and can select specific particles. Smaller particles (such as concentrate or pellets) can be consumed at a higher intake rate than longer particles (such as long, fibrous forages). Imbalanced nutrient intake and altered rumen fermentation as a result of sorting, can negatively affect digestion efficiency and production (36).

## Impact of Feeding Behavior on Productivity

Cattle feeding behavior has a significant impact on productivity (37, 38) and this is due to several reasons. First, an increase in the time spent eating facilitates chewing, reduces feed particle size, and increases digestibility (39). Second, a greater feeding time increases the production of saliva, which acts as a buffer over the rumen, decreasing acidity (40). It has also been suggested that by reducing the FR, the risk of metabolic problems such as sub-acute ruminal acidosis (SARA) and displaced abomasum is also reduced (41). In contrast, faster and more intense (high FR) meals facilitate the production of acid in the rumen, increasing the risk of acidosis (42, 43). Sorting behavior has also been identified as a behavior feature impacting productivity. Dairy cows receiving a mixed ration typically select in favor of short and fine particles and against longer forage ones (34, 44). This pattern may result in an imbalanced nutrient intake in relation to the formulated diet, having negative consequences on efficiency and production (45, 46). The combination of these (and possibly other) effects means that the impact of feeding behavior on productivity may be as important as feed intake. For instance, Shabi et al. (3) showed that the correlation between milk production (kg/day) and feeding behavior (time spent eating) is stronger (*r* = 0.4, *P* = 0.01) than the relationship between milk production and feed intake (*r* = 0.308, *P* = 0.05). Beyond milk production, feeding behavior has an effect on the milk quality, as demonstrated by the study of DeVries and Chevaux (47) in which there was a positive correlation between meal frequencies and the percentage of milk fat. Also, Macmillan et al. (48) found that an increase of feeding frequency increased milk fat. Other authors that have investigated this association could not confirm it; for example, Niu et al. (49) found no differences in milk fat composition between cows with high (2 times higher) feeding frequencies and cows with lower feeding frequencies. On the other hand, changes in feeding behavior are less likely to have an effect on milk protein as none of the previous studies found significant associations between the parameters (47–49). Based on the potential association between feeding behavior and milk quality, some studies have tried to implement dietary strategies to modify feeding behavior and improve milk quality traits. For instance, a study of grazing dairy cows in Chile found that supplementation with a flavor enhancer increased feeding time, FR and the ruminating time, which was associated with a higher milk production (kg) and percentage (%) of milk protein (50).

More efficient animals use less feed for maintenance, which increases the energy allocated to production (for example, growth or milk production). This not only leads to higher economic profitability but also to less waste products (manure, greenhouse gases, etc.) emitted to the environment (51, 52). Knapp et al. (53) reviewed a list of measures that may offer the possibility to reduce greenhouse gas (GHG) emissions from dairy cattle and the improvement of feed efficiency appeared among the top three. For instance, increasing milk production per feed unit will dilute the methane cost associated with maintenance energy requirements. Digestibility is a key feature that determines feed efficiency (54) and efforts are made to improve it, including changes in the feeding behavior, as reported in previously mentioned studies. The association between feeding behavior and feed efficiency has already attracted notable attention in beef cattle, where it has been shown that eating behavior has a profound impact on efficiency. In beef cattle, Robinson and Oddy (55) found that feed efficiency had phenotypic correlations of 0.64, 0.45, and 0.51 with the time spent eating, daily frequency of meals, and the number of visits to the feeder, respectively. Similarly, in growing dairy heifers, Green et al. (56) evidenced that feed efficiency, measured by the residual feed intake (RFI), was moderately to highly correlated with DMI (*r* = 0.54–0.74), indicating that with the same level of production (i.e., growth), efficient cattle ate less than cattle considered inefficient. When comparing less (the lowest 10%) and more (the highest 10%) efficient cattle based on RFI, it was shown that efficient animals ate less frequently but for longer, had lower FR and spend less time at the feeder compared to less efficient cattle. Indeed, some studies suggest that efficient animals tend to spend less time feeding (57) which may have evolved as a mechanism for energy conservation. Conversely, more efficient animals can spend more time resting, thus using less energy for activities other than eating.

It is well-known that feed efficiency increases when consumption decreases due to limited access to feed (58, 59). The reason is that a greater feed intake increases the passage rate that, in turn, decreases feed digestibility. This effect is especially pronounced when the diet contains a high percentage of fiber; this is because fiber digestibility is highly influenced by its passage rate through the rumen, which determines the accessibility of the fibrolytic microbiota to rumen content. These results suggest that feed efficiency can be improved by stimulating shorter but more frequent meals and lesser (or lower) FR.

In addition to feed intake, rumination is also a key feeding behavior trait for proper digestive functioning in cattle. Rumination aims to make feed (especially forage) more accessible for bacteria that facilitate the fermentation of fiber and increase its digestibility. However, the association between the time a cow spends ruminating and feed intake is controversial. Krause et al. (60) found that this association was positive (in long particle feed); whereas, Schirmann et al. (61) saw that cows that spent more time ruminating showed lower feeding times and less DMI, when calculated in 2-h feeding intervals. In general, though, feed consumption and rumination are mutually exclusive, which could explain this negative correlation. Rumination time may also have an impact on milk production, probably as a result of the positive association between rumination and lying times (62, 63). Soriani et al. (64) calculated this association and found that ruminating time is positively correlated (*r* = 0.36) with milk production (kg) during the first weeks of lactation. In short, the impact of rumination on performance suggest that production and efficiency are affected by not only the amount of feed eaten but also by the way that feed is consumed.

## Association Between Feeding Behavior and Welfare

**Figure 1:**
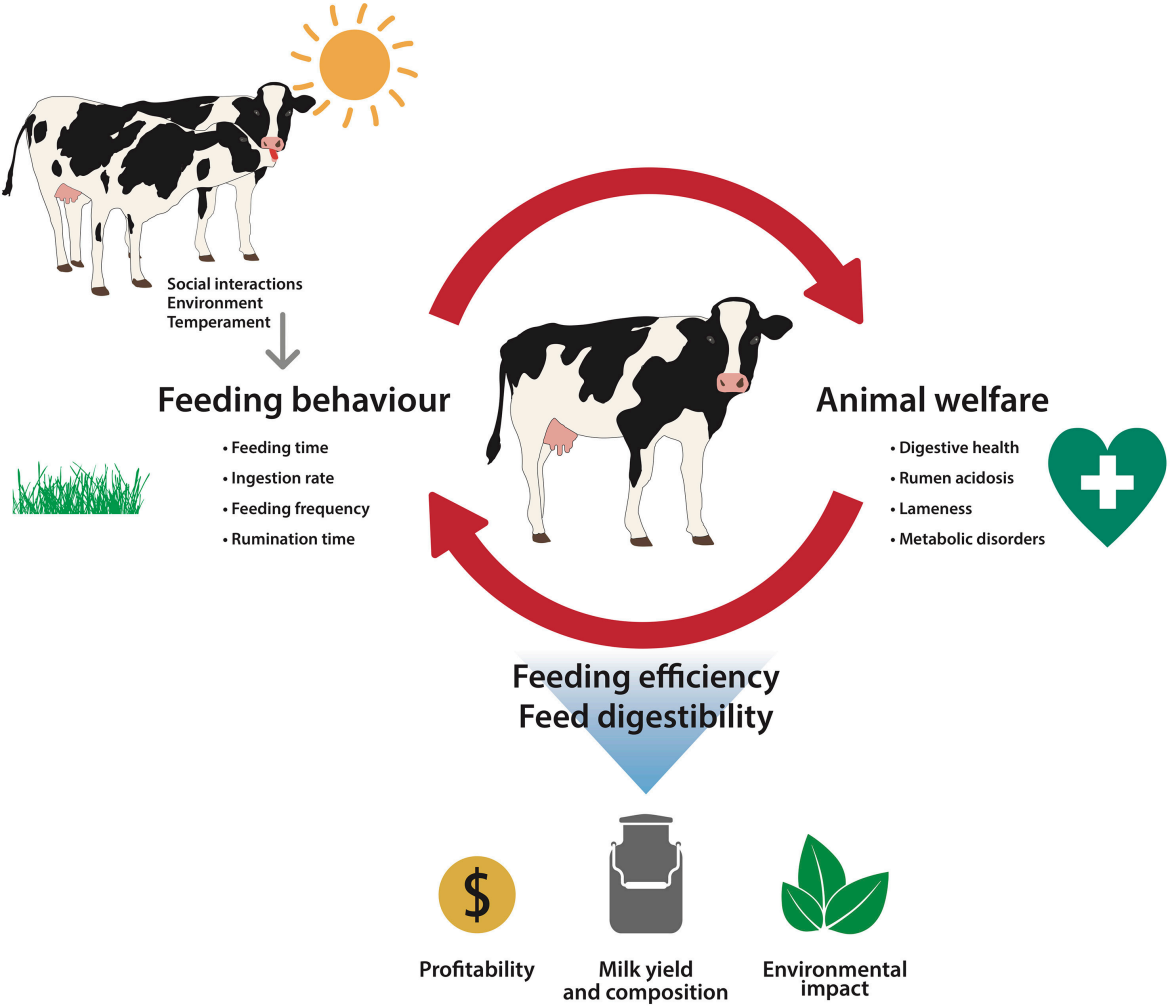
Outline of the bidirectional relationship between feeding behavior and animal welfare in dairy cattle, which includes the drivers of feeding behavior and the impact on productivity.

The study of feeding behavior has attracted considerable interest in recent years not only as a reflection of parameters such as efficiency or productivity, as described above, but also associated with welfare issues. The evidence suggesting a close association between feeding behavior and animal welfare is growing. For instance, the well-known principle of the Five Freedoms, include freedom from hunger (and thirst) as a capital aspect to safeguard welfare in farm animals (65). Additionally, this association is a bidirectional process in which feeding behavior impacts some aspects of cattle welfare but at the same time, the welfare status of cattle can also modify feeding behavior. Therefore, as represented in Figure 1, the cause-effect relationship between feeding behavior and welfare can be reciprocal.

### Feeding Behavior as a Consequence of Health and Welfare Problems

Any alteration of an animal's health status is often associated with changes in feeding behavior. In dairy cattle, this relationship has been used to identify health problems (32, 66). Previous studies are examples of how tools to monitor behavior, such as pedometers or electronic collars, can be used to identify sick animals in a quick and accurate manner. In the era of smart farming, this can be very valuable on dairies, as it may improve disease detection and facilitate adequate health treatments, which improves animal welfare and reduces production losses (67). The most widely known feeding behavior sign associated with illness is a decrease in feed intake. Some forms of illness likely reduce appetite, especially at the onset of disease. Fogsgaard et al. (68) reported that feeding behavior decreased when cows were suffering from mastitis. Huzzey et al. (69) found lower DMI during the first weeks post-partum for cows diagnosed with metritis. Bareille et al. (70) foresaw that clinical signs of lameness associated with hock and foot lesions, provoke a decrease in DMI of 5.6 and 6.4 kg at four and 5 days before it is diagnosed, respectively. This reduction in consumption is likely to decrease milk production by about 1.2 and 3.3 kg/day, respectively. The reason for this reduction in feed intake is that lameness is painful and lame cows are reluctant to move, even toward the feeding trough, causing a decrease in feed consumption (71).

In addition to feed intake, feeding pattern also changes during illness. Multiparous cows subsequently diagnosed with metritis or retained placenta tended to spend 5 or 10% less time feeding prepartum than healthy counterparts, respectively (72). According to Bareille et al. (70), cows diagnosed with ketosis reduced DMI by 7.5 Kg, on average, during lactation, which was associated with an average 45 min reduction of feeding time immediately before diagnosis. In 2010, von Keyserlingk and Weary (1) observed that, the week before calving, the likeliness of a cow to have severe mastitis increase 1.72 times per each 10-min decrease in feeding time and almost three times per each 1 kg decrease in DMI. Besides intake and feeding time, other studies have shown that an increase of FR is a consequence of illness and lameness, likely as a mechanism to maintain intake (32, 73). Schirmann et al. (61) suggested that when cows feel sick, they are less able to compete successfully for access to the feed bunk, and thus use higher FR when they have access. A notorious example of this relationship is the time spent eating in lame cows. The pain associated with lameness reduces the time spent standing, including standing at the trough, which reduces the time spent eating and increases the FR (74). For example, Bach et al. (75) observed a 268–240 min/day reduction in total time eating when comparing severely lame vs. healthy cows. Similarly, González et al. (32) suggested that some cows can increase FR by two to three times during acute or chronic lameness.

Rumination time is related to the resting time and the health status of cows (76, 77). A decrease in rumination time, combined with the onset of clinical disease, has been previously described for lameness (78), mastitis (68, 79), and metabolic disturbances (76, 80). For instance, Schirmann et al. (61) and Kaufman et al. (81) reported a decrease in rumination time for cows with subclinical ketosis during peripartum. Liboreiro et al. (82) confirmed this reduction from calving to 1 week postpartum for cows with subclinical ketosis. Changes in rumination time are also a reliable indicator of the time of calving, as there is a reduction of more than 50% of the time spent ruminating between 48 and 24 h before calving (64).

### Feeding Behavior as a Potential Cause of Welfare Problems

In addition to the causal relationship between health problems and changes in feeding behavior described so far, there are some cases where changes in feeding behavior driven by external factors can increase the risk of disease. A paradigmatic example is the relationship between time spent eating and ruminal acidosis. Daily feed intake determines the production of acid while chewing determines the production of saliva [which acts as rumen buffer (83)]. Therefore, these two components of feeding behavior (feed intake and chewing) during the day are closely related to pH and the acid-base balance in the rumen. Animals that are forced to spend less time feeding (by social competition, for example) often compensate for the time debit by eating faster. Such increases in FR may reduce salivation during feeding by 3–1 ml per g of DMI (84). Conversely, cattle with low FR chew feed more thoroughly; resulting in increased production of saliva, which increases the buffering capacity of the rumen. Thus, a greater time spent eating reduces the susceptibility to acidosis [both subclinical and clinical (85, 86)], and consequently, the occurrence of health problems associated with acidosis in dairy cows, such as laminitis and liver abscesses, would also be reduced (87).

Feed sorting is another example of feeding behavior that can result in health problems if certain conditions are met. Feed sorting in intensively fed lactating dairy cows may increase the risk of ruminal acidosis. Dairy cows receiving a TMR diet typically select short and fine particles (grain) and discriminate against longer forage ones (34, 33, 44). This pattern may result in a higher consumption of rapidly fermentable carbohydrates and lower effective fiber compared with the formulated diet, and it may result in lower ruminal pH (88). On the other hand, an increase in sorting for short and fine particles was associated with less time spent feeding (34), which has been previously identified as a risk factor for acidosis; nevertheless, in the same study feeding rate was positively correlated with sorting of long particles, and this observation disagreed with DeVries et al. (44), who observed that cows consume more quickly when receiving a ration containing a greater proportion of smaller particles. However, there are individual variations, with some animals being more susceptible to suffer ruminal acidosis than others, and these groups may have a different sorting pattern (89). These authors fed lactating dairy cows with high-grain diet and, depending on their acidosis index (severity of SARA), classified them in two groups, tolerant and susceptible animals to high-grain diet. Both groups sorted for short particles, but susceptible animals sorted to a greater extent. Also, susceptible animals sorted against long particles, whereas tolerant animals did not. Additionally, numerous studies suggest that typical patterns of feed sorting may be adjusted depending on the physiological status of the animal as a postingestive feedback mechanism (36). For example, various authors have noted alterations of feeding pattern in animals under ruminal acidosis, which modified typical preference for grain to more preference for long particles with higher content of physical effective fiber to attenuate the digestive upset (88, 90).

In brief, changes in feeding behavior may be both indicators and causes of health problems, especially for metabolic diseases such as ruminal acidosis and associated problems.

## Underlying Welfare Traits Affecting Feeding Behavior

Feeding behavior (regulated by the feedback between appetite and satiety) is the response to the homeostatic mechanism that motivates an animal to acquire nutrients. The duration of each meal would be combined with the signals that originate from the animal's energy balance (body composition) and the time the animal spent eating to determine when the next meal occurs. These dynamic processes can; however, be modulated by factors such as feed accessibility (feeding space per animal), dominance and hierarchy (influenced by a cow's weight, age and size, among other factors), an individual's temperament and that of the pen mates, and environmental conditions (such as temperature).

### Social Behavior and Temperament

Dairy cattle are social animals and are prone to establish dominance hierarchy, particularly when resources are limited such as at the feed bunk (91). The dominance rank determines the priority of access to resources such as feed (32). Dairy cattle reared in intensive systems have limited space availability per animal, which can exacerbate the effects of feed competition. In grazing systems, dominance also has an impact on feeding behavior and intake as dominant animals have priority access to the best quality feed (92). This could explain why dominant cows sometimes produce more milk than subordinate cows in grazing systems (93).

In intensive dairy systems, it is recommended that feeding space should be at least 0.6 m per dairy cow. However, even when space allowance is enough, some subordinate cows will still prefer to avoid proximity to dominant cows (94). Besides space availability, feed bunk design can also influence dominance behavior. According to Huzzey et al. (69), cows were displaced more frequently from a post-and-rail feed barrier, compared to a headlock barrier. Competition can increase in situations where a restricted feeding space converges with limited feed availability. This competition often results in shorter but quicker meals, increasing the FR and, consequently, the risk of acidosis in dairy cattle (95).

Social dominance is strongly correlated with a cow's age, body size, and seniority, which plays a key role in any existing, or newly formed, group of cows (96, 97). A higher dominance rank may lead to increased accessibility to feed with consequences on efficiency and productivity. In group housing, with no restrictions on accessing feed, dominants can often access feed more easily; whereas, subordinates may need to adapt their feeding behavior to accommodate bigger or more dominant animals. In dairy cattle, dominant animals displace subordinate cows from the feeder in order to show their preferred feeding patterns; whereas, subordinate animals must adjust their eating patterns in order to avoid conflicts with higher ranked cows (28, 93). Therefore, aggressive interactions and displacements at the feeder can influence feed intake, especially in subordinate cows. Galindo and Broom (98) identified a dominance trait (called displacement index), based on the number of times a cow displaces another, which determines the feeding time, showing that the most dominant individuals spend more time feeding. The positive impact of longer feeding times on productivity has already been mentioned in section Impact of Feeding Behavior on Productivity.

Social behavior can be managed through changes in husbandry and facilities, as feeding availability and space will affect social competition for feed. Considering the effects of hierarchy, limited access to feed or reduced intake by subordinate animals could reduce feed efficiency when compared to dominant cows with unlimited access to feed. The reason for such an effect, as explained above, is a decrease in feed efficiency when feeding duration is lower and FR is higher. The opposite effect has been shown in higher dominance rank cows. The additional time for feeding used by more dominant individuals can increase salivary secretion, reduce the size of feed particles (40) and therefore be beneficial to rumen fermentation (85). This is likely to result in improved nutrient digestibility and feed efficiency. The negative effects of social competition for subordinate cows can be mitigated by providing greater feeding space and increase the frequency of feed deliveries to reduce competition between pen mates (99).

Temperament can also have a significant effect on feeding behavior. The term temperament refers to individual differences in behavioral and physiological traits that are consistent over time and contexts. Some of the temperament traits most commonly studied in farm animals are fearfulness, reactivity, docility and aggression (100). Temperament in cattle is mainly assessed by short-term tests of behavior in response to a standardized stimulus, such as handling, isolation or exposure to a novel object. Animals that are more likely to cope with those situations are considered to have a “good” temperament. A study by Llonch et al. (101) on beef cattle observed that temperamental animals (e.g., steers that displayed a high flight speed response in a handling test) visited the feeder more often and spent, on average, less time eating per meal, eating less feed per meal also. The same authors hypothesized that, at the feeding area, most temperamental cattle are more reactive to the presence of other pen mates, increasing the likelihood of feeding interruption (101). This would inevitably lead to more frequent but shorter visits to the feeder. Temperament implications on feed efficiency and productivity have been studied. For instance, Nkrumah et al. (37) showed that more temperamental steers (i.e., animals showing a greater flight speed once released from the chute) had a lower ADG whereas Llonch et al. (102) revealed a decrease in feed intake in animals showing a higher flight speed. It is plausible that both results are related and the slower growth rate of temperamental cattle is caused by their smaller feed intake. However, it is still not clear whether this leads to a reduction in productivity and further studies are needed to confirm a possible association between temperament, feeding behavior and productivity in dairy cattle.

### Environmental Effects

One of the greatest threats for the welfare and productivity of dairy cattle associated with the environment is heat stress, caused by high environmental temperature and humidity. Heat stress is particularly relevant in dairy cattle since the effects on milk production and reproductive performance can be severe (103–105). The reduction in performance during heat stress has been commonly attributed to reduced DMI and a decreased availability of nutrients (103, 106). Changes in behavior and metabolic priorities markedly alters post-absorptive nutrient metabolism (107, 108). Heat stressed ruminants decrease their feed intake to reduce metabolic heat production and thus maintain a constant body temperature (109, 110). A consequence of this reduction in feed consumption is, among others, the decrease of milk production (103). High-yield milking cows are very sensitive to heat stress with negative consequences in milk yield, milk fat and protein content, and health (104, 111). In addition, dairy cows change their normal feeding pattern to eat when the temperature is lower, i.e., at dawn and dusk (112), and the eating time is concentrated in shorter periods of the day. In addition to the previous changes, cows will also show preferences to eat concentrated feed instead of fiber (109). This preference is due to a lower metabolic production of heat associated with concentrate feed compared to fiber-rich feed (113). Both the increase in frequency of meals during crepuscular times and the preference for concentrated feed (associated with a reduced forage intake), leads to an increased risk of ruminal acidosis (114, 115). On the contrary, heat stress can be linked to improved digestibility. The reason for this result is that the reduction in DMI decreases the rate of passage through the rumen (109, 116), and therefore increases the retention time (112) and improves the digestibility of structural carbohydrates (117).

There are different strategies to mitigate the effect of heat stress in cattle. These strategies include provision of shade (118), increased ventilation, and the combination of ventilation (fans) and showers (sprinklers) to increase heat loss from the body (103). However, there may be situations when that these strategies are insufficient to relieve heat stress. An alternative strategy to reduce the effects of heat stress is to provide moderate cold drinking water. In a study with dairy cows under high temperatures, where drinking water was refrigerated (from 28 to 10°C), Milam et al. (119) reported a decrease in body temperature, an increase in feed intake and a higher milk production in cows that received refrigerated water.

In addition to alleviating heat stress by decreasing the thermal load, there are strategies to compensate the changes in feeding behavior as a response to heat stress. This alternative can be developed when the options to mitigate heat stress are limited or simply unfeasible. To mitigate decreases in feed intake while sustaining milk production, farm managers will often increase the energy density of the diet by reducing the proportion of neutral detergent fiber on dry matter basis (120), increasing the proportion of digestive fiber (121) and increasing the concentrate portion of the diet (122). However, these strategies should be applied with caution as a higher carbohydrate digestibility can increase the risk of SARA. In addition, if the concentrate proportion is higher, the increased catabolism with elevated protein digestion can lead to increased rectal temperatures (123), suggesting that protein digestion elevates internal temperature. Therefore, a higher proportion of highly fermentable carbohydrates in the diet to counteract the reduction in feed intake can have downstream consequences for cow welfare. In beef cattle, Mader et al. (124) suggested that limit feeding in events of heat load can alleviate the metabolic load associated with rumination that aggravates the effects of high climatic heat load. Dietary treatments can also be used to modify feeding behavior in heat stressed cattle. For instance, Asparagus officinalis (125), conjugated linoleic acids (126), yeast cultures (127), niacin (128), and citrus extract (129) have been used to minimize, with more or less success, the effects of heat stress. However, most of them have inconsistent effects on feeding behavior and productivity and further research is needed to investigate the most effective heat stress dietary mitigation measures.

An extensive body of research has focused on how to mitigate the consequences of heat stress, especially from the production perspective. However, the reason why feeding behavior is affected during heat stress and how this can be tackled still needs to be addressed. Dietary treatments are promising strategies that can help modulate feeding behavior during heat stress, benefiting the animal's production requirements while prioritizing its optimal biological functioning.

## Concluding Remarks

Feeding behavior can be measured using different criteria such as the frequency and duration of meals, feeding rate (g/min) and rumination time. Higher DMI has been associated with a lower feed efficiency. However, it can improve if cows spend a longer time feeding, reducing the feeding rate. More frequent and shorter meals are associated with a more efficient use of feed; whereas, an increased rumination time usually results in greater milk production. Sorting behavior leads to imbalanced nutrient intake and altered rumen fermentation, resulting in impaired digestion efficiency.

Changes in feeding behavior can assist in identifying health problems such as acidosis or lameness. A reduction in rumination time is a reliable indicator of health problems. An increase in meal size or in sorting for small particles may increase the risk of ruminal acidosis, which can facilitate a higher incidence of lameness.

The natural behavior of cattle based on social hierarchy within groups has a significant impact on feeding behavior, especially in situations of limited access to feed. The competition for feed causes a reduction in average meal duration and increased FR (g/min). Dominant animals with *ad libitum* access to feed eat a larger amount of dry matter than subordinate individuals, causing a reduction in feed efficiency due to a decreased retention time. However, the opposite effect on feed efficiency is observed if they also spend longer time eating due to an increased production of saliva and the resulting improvement in feed digestibility. Providing sufficient space for cows to express feeding behavior at their will, can improve feed efficiency. Heat stress has a significant effect on feeding behavior as it reduces feed intake and alters the feeding pattern, increasing the risk of ruminal acidosis.

Research and innovation in feeding behavior can bring advances in animal welfare and production efficiency. Of especial relevance are feeding behavior strategies that can improve both the welfare of farm animals and their feeding efficiency, which inevitably reduces the impact of dairy cattle on the environment.

## Author Contributions

PL, GT, II, FB, and XM contributed to the concept of this paper. PL, MB, and GT carried out the literature search and compiled the articles. PL, EM, and MB wrote the manuscript. All authors contributed to the manuscript and approved the final version of the manuscript.

### Conflict of Interest Statement

II, FB, GT, and MB were employed by company Lucta S.A. The remaining authors declare that the research was conducted in the absence of any commercial or financial relationships that could be construed as a potential conflict of interest.
